# Deep Bayesian Gaussian processes for uncertainty estimation in electronic health records

**DOI:** 10.1038/s41598-021-00144-6

**Published:** 2021-10-19

**Authors:** Yikuan Li, Shishir Rao, Abdelaali Hassaine, Rema Ramakrishnan, Dexter Canoy, Gholamreza Salimi-Khorshidi, Mohammad Mamouei, Thomas Lukasiewicz, Kazem Rahimi

**Affiliations:** 1 Deep Medicine, Oxford Martin School, University of Oxford, Oxford, United Kingdom; 2 Department of Computer Science, University of Oxford, Oxford, United Kingdom

**Keywords:** Cardiovascular diseases, Computer science

## Abstract

One major impediment to the wider use of deep learning for clinical decision making is the difficulty of assigning a level of confidence to model predictions. Currently, deep Bayesian neural networks and sparse Gaussian processes are the main two scalable uncertainty estimation methods. However, deep Bayesian neural networks suffer from lack of expressiveness, and more expressive models such as deep kernel learning, which is an extension of sparse Gaussian process, captures only the uncertainty from the higher-level latent space. Therefore, the deep learning model under it lacks interpretability and ignores uncertainty from the raw data. In this paper, we merge features of the deep Bayesian learning framework with deep kernel learning to leverage the strengths of both methods for a more comprehensive uncertainty estimation. Through a series of experiments on predicting the first incidence of heart failure, diabetes and depression applied to large-scale electronic medical records, we demonstrate that our method is better at capturing uncertainty than both Gaussian processes and deep Bayesian neural networks in terms of indicating data insufficiency and identifying misclassifications, with a comparable generalization performance. Furthermore, by assessing the accuracy and area under the receiver operating characteristic curve over the predictive probability, we show that our method is less susceptible to making overconfident predictions, especially for the minority class in imbalanced datasets. Finally, we demonstrate how uncertainty information derived by the model can inform risk factor analysis towards model interpretability.

## Introduction

The application of deep learning to medicine has been growing over recent years. A lot of research in this field has been focusing on estimating and improving “point predictions” in form of personalized risk scores for a given medical event in one’s future, as for instance reported in innovative deep learning models such as RETAIN^1^ and Doctor AI^2^. However, point predictions in absence of uncertainty estimates lack credibility quantification and raise concerns about safety. Considering the significant consequences of decision making in clinical practice that is guided by model predictions, quantifying the uncertainty of predictions is proving to be a key step in putting these models to practice in medicine.

In the last several years, a new subfield of deep learning, called probabilistic deep learning, has drawn wide interest to provide probabilistic predictions and uncertainty estimations at the same time. The most promising methods are Bayesian deep learning (BDL)^3^ and sparse Gaussian processes (GP)^4^. In BDL, by placing a distribution over each of the model weights instead of treating them as point values, the uncertainty of the weights can be passed layer by layer to eventually estimate the uncertainty in the predictions. However, this approach usually requires a compromise between model complexity and expressiveness of variational distributions. On the contrary, the GP model, as a non-parametric model, is more flexible and expressive than BDL. This advantage comes, however, at the expense of the need to store and process the data points for the covariance matrix. This usually takes cubic time ${\mathscr {O}}(n^3)$ to calculate the inversion and determinant of the covariance matrix for inference^5^ and becomes a challenge when working with large-scale datasets. The state-of-the-art solution to this challenge is to use a small number of pseudo-points (i.e., inducing points) to approximate the data points. This enables the covariance matrix to be approximated by a lower-rank representation^6^. Since the entire dataset is summarized by a small number of inducing points, this method is called sparse GP, and its performance highly depends on the robustness of the inducing points and kernel parameters.Wilson et al.^7^ upgraded this framework to be more flexible and scalable by implementing a deep architecture beneath the kernel function as a feature extractor, which is known as deep kernel learning (DKL). Although the deep architecture provides a significant boost in representational power, the framework can only capture the uncertainty in the higher-level latent space (after the deep architecture). This results in a lack of interpretability of predictions, and failure to capture the uncertainty in the deep architecture.

The core idea of this paper is to combine the strengths of both frameworks by merging the BDL with the DKL framework. We expect that this can retain the expressiveness from GP while capturing the uncertainty during the feature extraction, leading to: (1) a stochastic representation from probabilistic feature extractor for more robust inducing points and kernel training; and (2) a more comprehensive uncertainty estimation. Additionally, we investigate how the uncertainty information naturally contained in the Bayesian components can contribute to an interpretable risk factor analysis in medical research.

## Background

### Ethics statement

Scientific approval for this study was given by the CPRD Independent Scientific Advisory Committee of UK (protocol number 16_049R). Data shared by consenting GP practices is de-identified and does not require individual patient consent for approved research (but individual patients can opt out from data sharing).

### Task description

**Figure 1:**
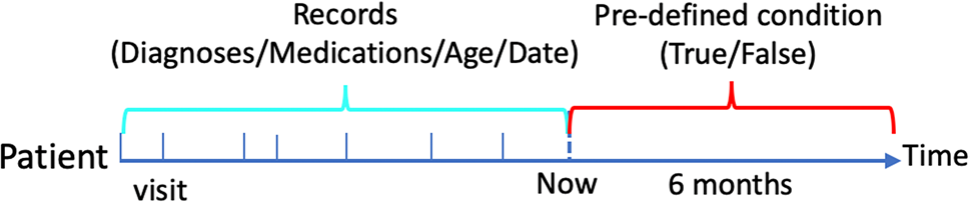
Illustration of prediction tasks. The axis represents time; medical records before ’now’ are used to predict the incidence of a condition in the following six months; the medical records include multiple visits, and each visit has one or more diagnoses and medications; the interval between two visits represents the time duration between them.

For this study, we explored a risk model for detecting the first incidence of three common chronic diseases, namely, heart failure (HF), diabetes and depression, using structured electronic health records (EHRs) from the Clinical Practice Research Datalink (CPRD). We used diagnoses (ICD-10), medications (British National Formulary code), event date (time stamp for each diagnosis and medication) and date of birth as historical medical trajectory to predict whether the first incidence of aforementioned conditions would be diagnosed in the following six months for a patient, and the conditions are treated as separate prediction tasks. The design is summarized in Fig. 1, and the ICD-codes for HF, diabetes, and depression are listed in the Supplementary. They were taken from previous publications^8,9^.

### Data source and cohort selection

CPRD is one of the most comprehensive de-identified longitudinal population-based EHR datasets. It contains primary care data collected from a network of general practitioner practices across the UK, and it is also linked to Hospital Episode Statistics and other health and area-based administrative databases. The data encompass 42 million patients, including 13 million currently registered patients. The patients included are nationally representative in terms of age, sex, and ethnicity^8^.

**Figure 2:**
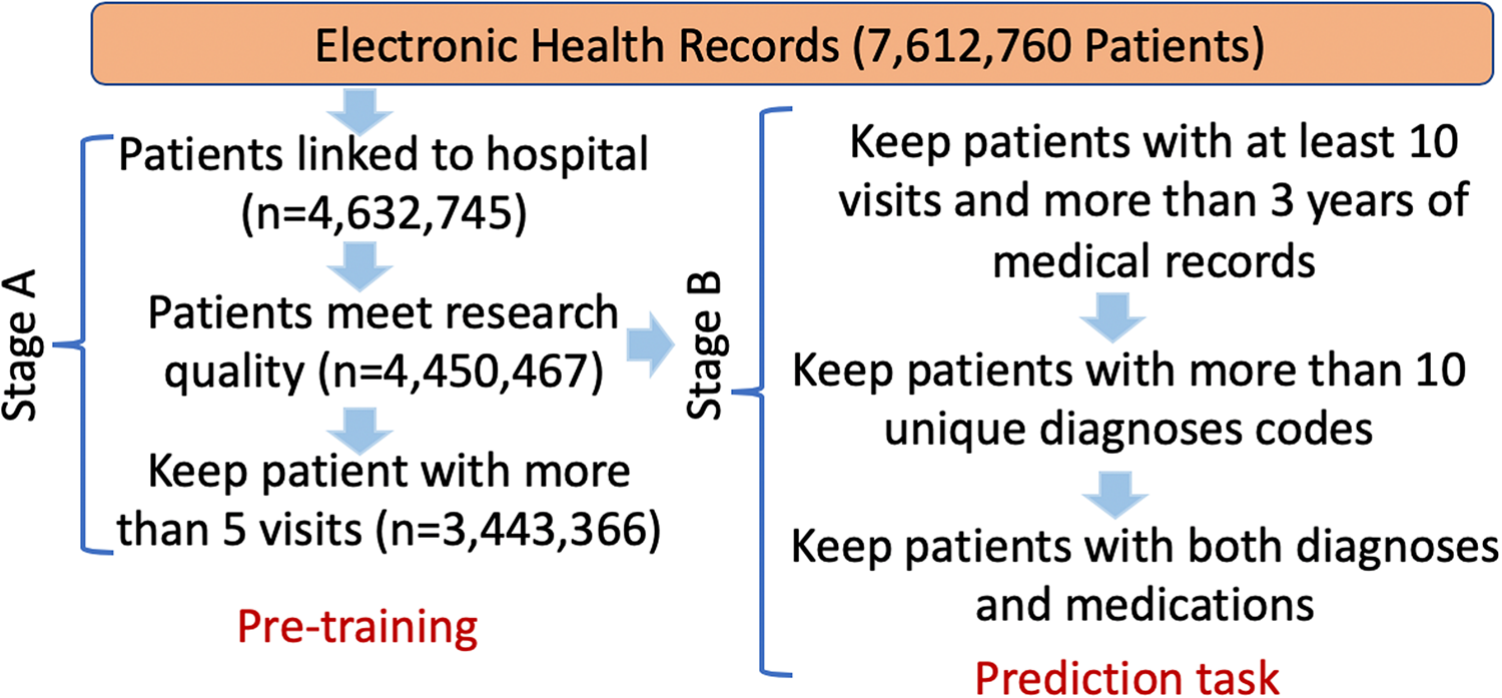
Cohort selection. Stage A illustrates the data cleaning pipeline from the raw CPRD dataset to the dataset for model pre-training, and research quality is an indicator provided by data provider. Stage B is used for patient selection for the incidence prediction tasks. The number of patients kept in each step is represented as n.

For this study, we set up a two-stage pipeline (A and B) for patient selection. Figure 2 illustrates the procedures and the number of patients kept within each step. Stage A was a general data linkage step to select patients that met the minimum requirements for the study. This dataset was used for general self-supervised pre-training. Stage B was designed for generating samples for the individual prediction tasks. Firstly, for a patient who had the pre-defined condition (HF, or diabetes, or depression), records were formatted as in Fig. 1, where all medical records before the first incidence of the condition were included as history records. For a patient who did not have the pre-defined condition, we randomly selected a time point to separate the records into history records and marked the patient as a negative sample. For all negative patients, we made sure that they had more than six months medical records after the selected time point to guarantee each of them was an absolute negative sample. Therefore, avoiding any assumption for the unseen future. The patient selection rules in stage B that kept patients who had enough records to be trained, eventually led to 788,880 (8.3% positive samples), 913,799 (11.3% positive samples) and 1,453,012 (16% positive samples) patients for HF, diabetes and depression, respectively. We refer to the datasets from stages A and B as datasets A and B, respectively.

## Related work

### BEHRT

**Figure 3:**
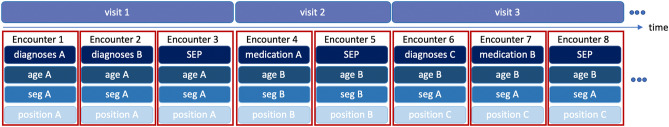
Illustration of four feature layers. Clinical diagnoses/medications, age, segmentation and positional code are included as features; each visit could have multiple encounters, and each encounter is a representation of multiple feature layers; summation of all embeddings are used for encounter representation.

BEHRT^10^ is a recently developed model that applied the concept of self-attention Transformer from natural language processing to EHRs. BEHRT took advantage of the self-attention mechanism and sequential format of EHRs in a way that maximally preserves the EHR-like structure. The feature structure is shown in Fig. 3, with each encounter corresponding to the so-called ’token’ in Transformers^11^. More specifically, by depicting diagnoses/medications as words, visits as sentences, and a patient’s entire medical history as document, BEHRT managed to model EHRs in the same way BERT^11^ modelled texts. Furthermore, with age, segment, and position as additional features, BEHRT was able to represent sequential information in EHRs in several different manners. As Fig. 3 shows, there are four embedding matrices for diagnoses and medications, age, segmentation and position separately. BEHRT uses the summation of the embeddings to represent each encounter. Recent work has shown that BEHRT outperforms other deep learning models for disease prediction based in the context of complex large-scale sequential EHR. More detailed information can be found in the original paper^10^.

### Introduction of uncertainty

The uncertainty in modelling can be divided into three categories: aleatoric uncertainty, epistemic uncertainty, and predictive uncertainty. Aleatoric uncertainty refers to the notion of randomness, and it usually indicates the uncertainty in the data^12^. Epistemic or model uncertainty refers to the uncertainty in the structure and parameters of a model caused by a lack of knowledge^12^. Predictive uncertainty is the uncertainty of prediction (e.g., for a sample or an individual). In this work, confidence (mean) and predictive uncertainty (standard deviation) are two variables to describe the predictive distribution for a patient, and hence are highly correlated.

### Gaussian processes

GPs are expressive non-parametric models^13^ with natural properties for uncertainty estimation. We only consider regression at this stage, but they can be easily used for binary classification tasks by wrapping a logistic regression^14^. If we have observed training data, ${\mathscr {D}}=\{x_{i}, y_{i}\}^N_{i=1}$ with $x_{i} \in \chi$ and $y_{i} \in {\mathbb {R}}$, our target is to predict an output $y^{*}$ for new inputs $x^{*}$. GPs usually place a GP prior over the latent function as $f \sim {\mathscr {G}}{\mathscr {P}}(v(\cdot ), k(\cdot , \cdot ))$, where $v:\chi \rightarrow {{\mathbb {R}}}$ is the mean function, and it is often taken as zero. The kernel function $k:\chi \times \chi \rightarrow {{\mathbb {R}}}$ controls the smoothness of GPs. A likelihood is then used to relate the latent function to the observed data through some noise, which is represented as $y_{i}=f(x_{i})+\epsilon _{i}$, $\epsilon _{i}\sim {\mathscr {N}}(0, \sigma ^2_{noise})$. In the end, we use the posterior for predictions and the marginal likelihood for selecting hyperparameters, which is shown in Equation 1:

1 $$\begin{aligned} \log \,p(y)=-\frac{1}{2}\varvec{y^T}\varvec{K^{-1}_{n}}{\varvec{y}}-\frac{1}{2}\log |\varvec{K_{n}}|-\frac{N}{2}\log (2\pi ), \end{aligned}$$

where $$\varvec{K_{n}}=\varvec{K_{\text {f}\text {f}}}+\sigma ^2_{noise}{\varvec{I}}$$ and $$\varvec{|K_{\text {f}\text {f}}|}_{i,j}=k(x_{i},x_{j})$$.

### Sparse-GPs

Because of the complexity of calculating the determinant and inverse, many approaches have been proposed to approximate $\varvec{K_{\text {f}\text {f}}}$ with a lower rank matrix^4^. One popular approach is posterior approximation through variational free energy proposed by^15^. The method suggests to optimize the evidence lower bound to minimize the KL divergence between posterior and variational distribution. Therefore, it directly approximates the posterior with a relatively small number of inducing points, and eventually simplifies the calculation. The evidence lower bound is shown as below:

2 $$\begin{aligned} \begin{aligned} {\mathscr {L}}_{Sparse-GP}=&-\frac{1}{2}\varvec{y^T}\varvec{Q^{-1}_{n}}{\varvec{y}}-\frac{1}{2}\log |\varvec{Q_{n}}|-\frac{N}{2}\log (2\pi )-\frac{t}{2\sigma ^2_{noise}} \end{aligned}, \end{aligned}$$

where $\varvec{Q_{n}}=\varvec{Q_{\text {f}\text {f}}}+\sigma ^2_{noise}{\varvec{I}}$, $\varvec{Q_{\text {f}\text {f}}}=\varvec{K^T_{\text {u}\text {f}}K^{-1}_{\text {u}\text {u}}K_{\text {u}\text {f}}}$, $t=Tr(\varvec{K_{\text {f}\text {f}}}-\varvec{Q_{\text {f}\text {f}}})$, $\varvec{[K_{\text {u}\text {f}}]}_{m,i}=k(z_m,x_i)$, $\varvec{[K_{\text {u}\text {u}}]}_{m,i}=k(u_m,u_i)$, $U=\{u_m\}^M_{m=1}$ represents inducing points.

### KISS-GPs

Besides posterior approximation^16^, proposed a structured kernel interpolation framework to produce a more scalable kernel approximation, named KISS-GP. This method combines structure-exploiting approaches, inducing points and sparse interpolation to further reduce the inference time cost and storage costs from ${\mathscr {O}}(M^2N+M^3)$ and ${\mathscr {O}}(MN+M^2)$ in Gaussian processes, respectively, for sparse-GPs to ${\mathscr {O}}(N)$. The main idea of this method is to impose the grid constraint on the inducing points. Therefore, the kernel matrix $\varvec{K_{\text {u}\text {u}}}$ admits the Kronecker structure or a Toeplitz covariance matrix for a much easier calculation. For the cross kernel matrix $\varvec{K_{\text {u}\text {f}}}$, it can be approximated, for example, by a local linear interpolation with adjacent grid inducing points as shown below:

3 $$\begin{aligned} k(x_{i}, u_{j})\approx w_{i}k(u_{a}, u_{j})+(1-w_{i})k(u_{b},u_{j}), \end{aligned}$$

where $u_{a}$ and $u_{b}$ are two inducing points on the grid that are closest to $x_{i}$, and $w_{i}$ is an interpolation weight that represents the distance to the inducing point. Eventually, the $\varvec{Q_{\text {f}\text {f}}}$ matrix in Eq. 2 can be approximated as Eq. 4:

4 $$\begin{aligned} \varvec{Q_{\text {f}\text {f}}}\approx {\varvec{W}}^T_{\text {u}\text {f}}{\varvec{K}}_{\text {u}\text {u}}{\varvec{W}}_{\text {u}\text {f}} \end{aligned}$$

### Deep kernel learning

In addition to sparse GP^7^, moved one step further and proposed to embed deep neural networks (DNNs) with GPs to learn more flexible representations. The kernel function transforms $k(x_{i},x_{j}|\varvec{\theta })$ to $k(g(x_{i},{\varvec{w}}),g(x_{j},{\varvec{w}})|\varvec{\theta },{\varvec{w}})$, where $\varvec{\theta }$ are the kernel hyperparameters, $g(\cdot )$ is a non-linear DNN, and ${\varvec{w}}$ are the parametrized weights of the network. Therefore, the DNN acts as a feature extractor to represent samples as latent vectors, and GPs can make inferences based on the learned latent features.

### Variational inference for Bayesian deep learning

Bayesian deep learning is another approach to make feature extraction and/or inference with uncertainty estimation. Instead of using point weights as deterministic DNNs, it places distributions over all model parameters, and the predictive distribution can be estimated by marginalizing the parameters^17^; this is shown as Eq. 5.

5 $$\begin{aligned} p(y^*|x^*,{\mathscr {D}})=\int _\Omega p(y^*|x^*,{\varvec{w}})p({\varvec{w}}|{\mathscr {D}})d{\varvec{w}}, \end{aligned}$$

where ${\varvec{w}} \in \Omega$ represents parameterized weights. However, the posterior $p({\varvec{w}}|{\mathscr {D}})$ is usually intractable in neural networks. In order to retrieve it^18^, proposed to approximate the posterior by optimizing the evidence lower bound to minimize the KL divergence between variational distribution and posterior, which is shown in Eq. 6:

6 $$\begin{aligned} \begin{aligned} {\mathscr {L}}_{BDL}=&\int _\Omega q_{\varvec{\gamma }}(w)log(p({\mathscr {D}}|{\varvec{w}}))d{\varvec{w}}-KL(q_{\varvec{\gamma }}({\varvec{w}})||p({\varvec{w}}))\quad , \end{aligned} \end{aligned}$$

where $q_{\varvec{\gamma }}$ represents the variational distribution, which is parameterized by $\varvec{\gamma }$. Afterwards, $p({\varvec{w}}|{\mathscr {D}})$ in Equation 5 can be replaced by $q_{\varvec{\gamma }}({\varvec{w}})$ for inference.

### Challenges in uncertainty evaluation

Quantifying the quality of uncertainty estimation is still an open question. One potential way to properly assess the correctness of the posterior is to compare it with the ground truth, and it is usually established using Hamiltonian Monte Carlo (HMC). However, HMC scales poorly on high dimensional space, thus, it is only feasible to a relatively simple model. In recent research, the test log-likelihood has gained wide popularity to be used to indicate the model’s credibility to capture the true posterior^19,20^. However^21^, did an experiment to compare the approximated posteriors from inference methods such as probabilistic backpropagation, matrix-variate Gaussian and Bayes by hypernet with the ’ground-truth’ established using HMC. Even though the approximate posteriors incorrectly had a lower variance, they still yielded a similar test log-likelihood as the ground-truth. Therefore, we argue that the test log-likelihood would be more meaningful to evaluate the posterior mean rather than the uncertainty (variance), and it is not a reliable criterion for determining how well an approximate posterior aligns with the true posterior. Therefore, we will discuss two qualitative criteria that we use for uncertainty evaluation in the following sections.

## Proposed methods

### Deep Bayesian Gaussian processes

In this section, we present the approach of our work, which combines DKL with Bayesian inference for a more robust uncertainty estimation. More specifically, DKL is used with a neural network as a feature extractor. The weights of the neural network are made stochastic by deploying a Bayesian deep learning framework. Because the probabilistic feature extractor can provide unlimited representations, it reinforces the training for inducing points and kernel parameters of sparse Gaussian processes as adding noises or augmentations to the representation^22,23^. Also, it propagates the uncertainty information through the entire model, which leads to more comprehensive predictive uncertainty estimation. We refer to this architecture as deep Bayesian Gaussian processes (DBGPs). Additionally, we show how to learn the properties of these kernels as part of a scalable GP. We start with the kernel and inference of a GP, and the kernel hyperparameter $\varvec{\theta }$ is ignored for the following parts to simplify the illustration. As for Eq. 3, the base kernel is shown as $k(x_{i}, x_{j})$; thus, the inference stage can be represented as:

7 $$\begin{aligned} p({\varvec{f}}^*|{\varvec{x}}^*,{\mathscr {U}},{\mathscr {D}})= & {} \int p({\varvec{f}}^*|{\varvec{x}}^*,{\varvec{f}}_{m}, {\mathscr {U}})p({\varvec{f}}_{m}|{\mathscr {D}},{\mathscr {U}})d{\varvec{f}}_m, \end{aligned}$$

8 $$\begin{aligned} p({\varvec{y}}^*)= & {} \int p({\varvec{y}}^*|{\varvec{f}}^*)p({\varvec{f}}^*)d{\varvec{f}}^*, \end{aligned}$$

where ${\varvec{f}}_m$ represents the latent prior from inducing points for sparse GPs, and ${\mathscr {U}}$ represents inducing points. For DKL, the kernel is transformed to $k(g(x_{i},{\varvec{w}})$, $g(x_{j},{\varvec{w}}))$, such a kernel function is used to measure the similarity between two latent representations extracted by a DNN. Accordingly, the inference for $p({\varvec{f}}^*)$ is changed to Equation 9, while $p({\varvec{y}}^*)$ remains the same.

9 $$\begin{aligned} p({\varvec{f}}^*|{\varvec{x}}^*,{\mathscr {D}}, {\mathscr {U}},{\varvec{w}}) = \int p({\varvec{f}}^*|{\varvec{x}}^*, {\varvec{w}},{\varvec{f}}_{m}, {\mathscr {U}})p({\varvec{f}}_{m}|{\mathscr {D}},{\mathscr {U}})d{\varvec{f}}_m \end{aligned}$$

In addition to DKL, the weights ${\varvec{w}}$ in $g(x_{i},{\varvec{w}})$ are also made stochastic by deploying a Bayesian deep learning framework in DBGP. Therefore, besides marginalizing the ${\varvec{f}}_{m}$, we also need to marginalize the weights for the inference, and this can be approximated using Markov chain Monte Carlo. The $p({\varvec{f}}^*)$ for DBGP is now transformed as follows:

10 $$\begin{aligned} \begin{aligned} p({\varvec{f}}^*|{\varvec{x}}^*,{\mathscr {D}}, {\mathscr {U}}) = \int _{\varvec{\Omega }}\int p({\varvec{f}}^*|{\varvec{x}}^*, {\varvec{w}},{\varvec{f}}_{m}, {\mathscr {U}})p({\varvec{w}}|{\mathscr {D}}) p({\varvec{f}}_{m}|{\mathscr {D}},{\mathscr {U}})d{\varvec{f}}_m d{\varvec{w}}. \end{aligned} \end{aligned}$$

Since the posterior $p({\varvec{w}}|{\mathscr {D}})$ is usually intractable, we use a variational distribution $q_{\varvec{\gamma }}({\varvec{w}})$ (e.g., mean field distribution^3^) parametrized by $\varvec{\gamma }$ to approximate it and then jointly train all the kernel hyperparameters {$\varvec{\theta }, \varvec{\gamma }$} together by optimizing the evidence lower bound and update the hyperparameters. Here, $\varvec{\gamma }$ and $\varvec{\theta }$ represent parameters relate to the feature extractor and kernel function (e.g., radial basis function (RBF) kernel and inducing points) respectively. In this work, we will mainly focus on GPs, but a more general family of elliptical processes (e.g. Student-t processes) can also be considered.

**Figure 4:**
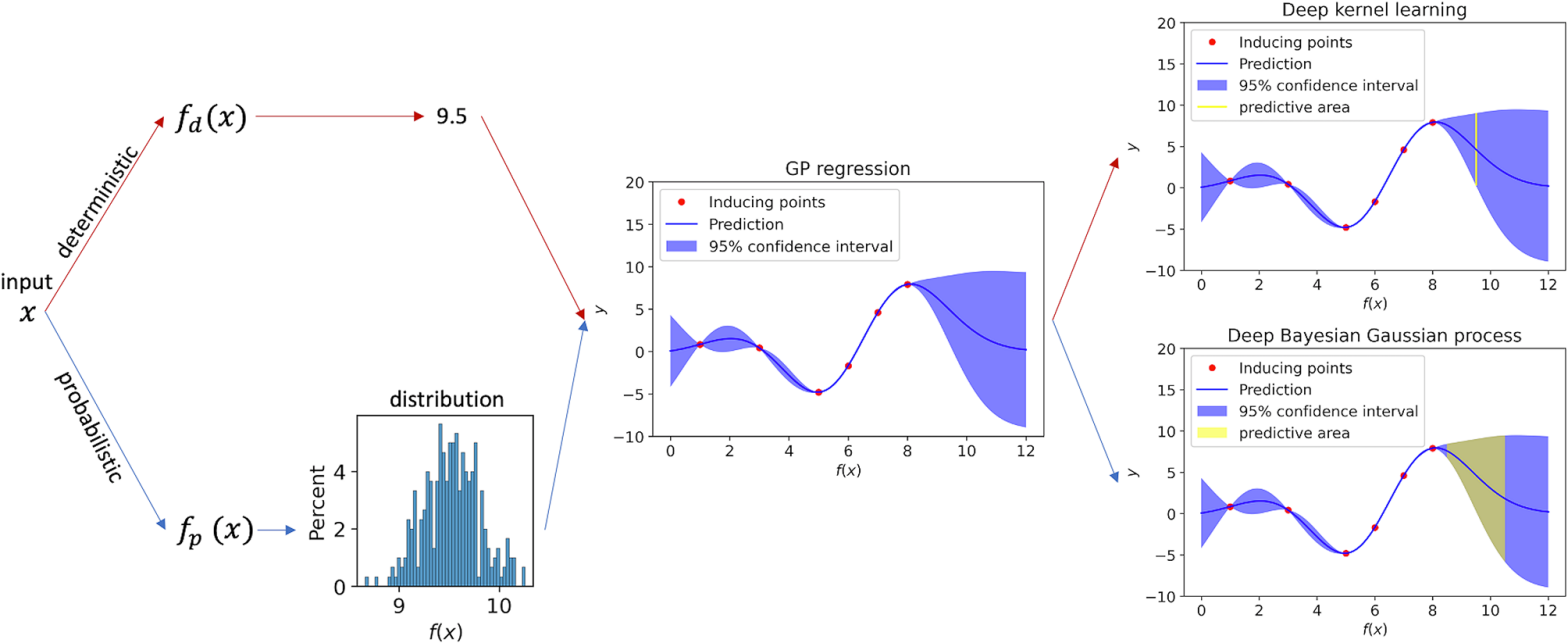
Predictive coverage for DKL and DBGP. The red path and the blue path represent DKL and DBGP, respectively. DKL maps the raw input *x* to the latent features, which is 9.5 in the figure, using a deterministic model $f_{d}$. Thus, the GP regressor predicts *y* for the given $f_{d}(x)=9.5$, and the potential prediction *y* is represented as the yellow line. In contrast, DBGP maps the input *x* to the latent distribution using a probabilistic model $f_{p}$, and the GP regressor predicts *y* conditioned on the latent distribution $f_{p}(x)$. The potential prediction *y* is represented by the yellow area. Typically, DKL and DBGP make prediction by marginalising the yellow line and the yellow area, respectively. Only one GP regressor is shown here to simplify the description. In practice, both GP regressor and $f_{p}$ or $f_{d}$ are trained together.

An illustration of the conceptual difference between DKL and DBGP in terms of the predictive coverage is shown in Fig. 4. Unlike DKL, which maps the raw features into a fixed latent representation (*x* in the figure) by a deep architecture, where the uncertainty estimation of *f*(*x*) completely comes from the GP, DBGP is able to capture the uncertainty hierarchically. The uncertainty captured by the deep Bayesian architecture is firstly reflected on the uncertainty in the latent representation (*x* in the figure). Afterwards, such uncertainty in the latent representation moves forward to the GP. Eventually, the predictive uncertainty represents the uncertainty in both *x* and *f*(*x*) dimensions, which is more comprehensive and shows a wider predictive coverage. Additionally, another benefit in this framework is for inducing points training. Because sparse GPs summarize the entire dataset into a small number of inducing points, the robustness of these inducing points directly decides the performance for inference. In DBGP, a stochastic feature extractor provides unlimited representations, and it can be considered as adding noises and augmentations to the representations. Therefore, we would expect the inducing points can be trained better than deterministic feature extractor in DKL^22,23^, leading to better generalizability and uncertainty estimation.

Although the standard GPs are discussed here, both DKL and the proposed framework (i.e, DBGP) are also compatible with other GP-related methods, such as deep Gaussian processes^24,25^. Deep Gaussian processes use variational approximation to build hierarchical stacked GPs. They overcome the drawback of having limited kernel expressiveness in the standard GPs. Therefore, deep Gaussian processes can be more flexible classifiers and regressors than the standard GPs.

### From regression to classification

The GP regression and GP classification share similar fundamental approaches. Instead of producing a continuous estimation with mean and variance that follow a Gaussian distribution, GP classification further produces an output to represent the probability of belonging to class 1 for a given input using a transfer function $\pi$. Typically, for a binary classification problem, the transfer function $\pi$ is a logistic function.^26^ Thus, there are two steps to transfer GPs from regression to classification: (1) computing distribution of latent function as Equation 7, (2) using logistic function over the latent function to produce a probabilistic prediction as Equation 11^13^, where $\pi ({\varvec{f}}^*)=p({\varvec{y}}^*=1|{\varvec{f}}^*)$. The error in GP classification is estimated using Bernoulli likelihood.

11 $$\begin{aligned} p({\varvec{y}}^*=1) = \int \pi ({\varvec{f}}^*)p({\varvec{f}}^*)d{\varvec{f}}^*. \end{aligned}$$

### Experimental setup

**Figure 5:**
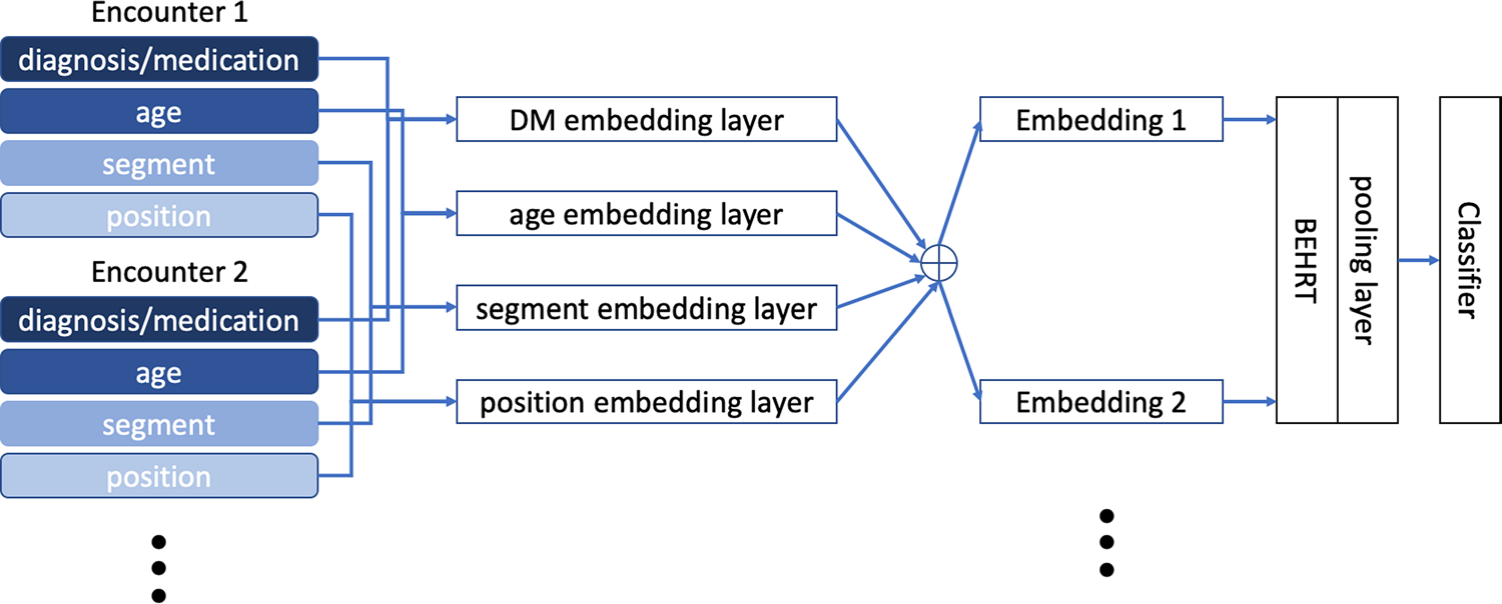
Model architecture. The model includes an embedding layer to encode the input features, a feature extractor based on BEHRT, and a classifier for prediction. DM represents diagnosis and medication; the pooling layer only extracts the latent representation of the first encounter (time step) for classification.

Figure 5 shows the fundamental model architecture that is used in the experiments to compare among three different frameworks: DBL, DKL, and the proposed DBGP. It includes a BEHRT as the feature extractor to map the raw EHRs to the high-dimensional latent representations. Afterwards, the pooling layer extracts the representation of the first time step from BEHRT for classification. The BEHRT model shares the same architecture as the original paper^10^ except it is a deterministic model in the original paper. The modifications that transfer BEHRT to a probabilistic model, as well as the definitions of different architectures are listed below:

- *Bayesian Embedding + KISS-GP (DBGP)* The proposed DBGP framework with BEHRT as a feature extractor, in which the embedding parameters are stochastic, and a KISS-GP as the classifier; all the other parameters are deterministic.
- *Bayesian Embeddings (BE)* BEHRT with a linear classifier in which the embedding parameters are stochastic, and all the other parameters are deterministic.
- *Bayesian Output (BO)* BEHRT with a linear classifier in which the classifier parameters are stochastic, and all the other parameters are deterministic.
- *Bayesian Embedding + Output (BE + BO)* BEHRT with a linear classifier in which the embedding and classifier parameters are stochastic, and all other parameters are deterministic.
- *Sparse-GP* A BEHRT model with a sparse-GP as the classifier; all the other parameters are deterministic.
- *KISS-GP* A BEHRT model with a KISS-GP as the classifier; all the other parameters are deterministic.

Dusenberry et al.^27^ indicate that for BDL, models with Bayesian embedding and Bayesian output usually work better than fully Bayesian models, so we mainly investigate the Bayesian model with stochastic embedding and output.

Models are implemented in PyTorch and GPyTorch^28^. The feature extractor, BEHRT, uses maximum sequence length 256, hidden size 150, 4 layers of Transformer. For each Transformer layer, we use 6 attention heads, 108 intermediate hidden size, and 0.29 dropout rate. For BE, BO, BE+BO, 150 is used for pooling layer, while 24 is used for sparse-GP, KISS-GP, and DBGP. The classifier is either a GP classifier or a feed-forward layer with logistic function for binary classification. For stochastic weights, we used mean field distribution as variational posterior, where each weight is represented by a normal distribution with learnable mean and diagonal variance parameters. Normal distributions with zero mean and 0.374 standard deviation are used as priors. For all GP components, 40 inducing points and RBF kernels are used, and they are implemented with a multivariate distribution with zero mean and identity covariance matrix for the prior. These parameters are selected with Bayesian hyperparameter optimization. To train Bayesian deep learning models and DBGP, we used batch size 64 and Adam optimizer^29^ with learning rate 3e-5 and weight decay 0.01. Bayes by Backprop^18^ with Blundell^30^ KL weight penalty are used for training.

### Evaluation methods

In this work, MC^17^ was used for all probabilistic models to estimate the predictive distribution, and we evaluated the model performance from three perspectives: generalization, ability of rejecting overconfident predictions and uncertainty estimation. Here, rejection ability refers to avoiding making overconfident predictions rather than making wrong predictions when the model performance is poor. For generalization, we evaluated the area under the receiver operating characteristics (AUROC) curve and the average precision (AP)^31^; both of them were calculated based on the mean predictive probability, which is a probability averaged over samples sampled from the predictive distribution. As for the ability of rejecting overconfident predictions, we evaluated the accuracy and AUROC as a function of the mean predictive probability. In medicine, it is highly desirable to avoid overconfident and incorrect predictions. Therefore, it is more useful to evaluate the model performance for predictions above a user-specified threshold. One tends to trust the predictions more when the confidence is high, and resorts to a different solution when the prediction is not confident. Thus, the better rejection ability can be directly reflected by having a higher performance for high-confident predictions. For uncertainty estimation, we propose (1) to treat the uncertainty measurement for classification differently by measuring the difference between the variance of true positives(TP)/negatives(TN) and the variance of false positives(FP)/negatives(FN). We would intuitively expect that the variance of TP/TN is distinguishably lower than the one of FP/FN. On the contrary, if the variance for both TP/TN and FP/FN are similar, we would say the model cannot provide a meaningful uncertainty estimation; (2) for imbalanced datasets, because the model is usually biased by the majority class, we would intuitively expect the model to have a higher uncertainty for the minority classes. Therefore, these two criteria are used as indication of the quality of the uncertainty estimation. To quantify the performance of the former criteria, we propose to calculate the Kullback-Leibler (KL) divergence between the uncertainty (standard deviation (std)) distribution of TP/TN and the uncertainty (std) distribution of FP/FN as Equation 12, and the larger the value, the better the performance. We can assume that both distributions are Gaussian distributions because of the central limit theorem^32^.

12 $$\begin{aligned} DIV = KL(p(F)||p(T)). \end{aligned}$$

Here KL represents the KL divergence, *p*(*F*) is the distribution of uncertainty for FP/FN, and *p*(*T*) is the distribution of uncertainty for TP/TN.

### Uncertainty analysis in embeddings

In addition to the model performance evaluation, for DBGP with stochastic embeddings, we explore the linkage between embedding uncertainty and relative risk associations by summarizing the entropy across all embedding dimensions. For diagnoses or medications with high uncertainty in the embeddings, we assume they would contribute more uncertainty to the latent representations as well as the predictions. Therefore, higher uncertainty in the embedding indicates an unclear association between a disease or a medication and the target disease, otherwise, the association would be more certain even though the magnitude of the association is strong or weak. We used the summation of entropy^33^ across the embedding dimension to indicate the embedding uncertainty, and a detailed discussion will be covered in Section .

## Results

We used the MC method to sample each patient’s prediction 30 times as an estimation of the predictive distribution for all analyses within this section. For comparison, we repeated the analysis with 60 samples and found no material difference in the prediction, and the results can be found in the Supplementary.

### Generalization performance

We compared the performance for generalization of aforementioned six probabilistic models by validating models on an external hold-out validation dataset. In our experiments, we split each dataset from stage B (HF, diabetes and depression) into 70% training set and 30% validation set. For the 70% training set, 50% and 20% are used for training and model tuning, respectively. After model tuning, we re-train model with all samples in the 70% training set and evaluate the performance on the validation set. Before training for a specific prediction task, we pre-trained the original deterministic BEHRT model on the dataset A, which was explained in Section , based on an self-supervised masked language model task^11^. Then, to fine-tune the model for the prediction task, we initialized the deterministic parameters with the pre-trained model parameters, and for all stochastic components, we used the pre-trained parameters as the mean of their variational distribution. Since the classifier was not a part of the masked language model training task, all the parameters within the classifier were randomly initialized. Table 1 demonstrates the performance for the marginalized prediction performance of each probabilistic model. It shows that all implemented probabilistic models have a comparable performance in terms of AUROC and AP. Heart failure has better performance than diabetes, and depression has the worst performance among these three prediction tasks. The result is expected, because all models share the same fundamental BEHRT model.

**Table 1:** Metrics for marginalized predictions on heart failure, diabetes, and depression. 95% confidence intervals are computed via a validation set bootstrapping with 50 bootstrap sets.

Model	HF		Diabetes		Depression	
	AUROC	AP	AUROC	AP	AUROC	AP
DBGP	0.941 (± 1e−3)	0.625 (± 5e−3)	0.834 (± 2e−3)	0.533 (± 4e−3)	0.776 (± 2e−3)	0.416 (± 3e−3)
Sparse-GP	0.945 (± 1e−3)	0.645 (± 2e−3)	0.834 (± 1e−3)	0.538 (± 3e−3)	0.782 (± 1e−3)	0.433 (± 2e−3)
KISS-GP	0.945 (± 1e−3)	0.649 (± 5e−3)	0.837 (± 2e−3)	0.538 (± 4e−3)	0.782 (± 1e−3)	0.433 (± 2e−3)
BE	0.942 (± 1e−3)	0.631 (± 7e−3)	0.830 (± 1e−3)	0.529 (± 2e−3)	0.774 (± 1e−3)	0.409 (± 2e−3)
BO	0.933 (± 1e−3)	0.645 (± 6e−3)	0.825 (± 1e−3)	0.525 (± 5e−3)	0.765 (± 1e−3)	0.425 (± 4e−3)
BE+BO	0.941 (± 1e−3)	0.628 (± 5e−3)	0.835 (± 2e−3)	0.538 (± 2e−3)	0.778 (± 1e−3)	0.419 (± 1e−3)

*AP* average precision.

### Accuracy and AUROC as a function of confidence

We re-used the results from the experiments in the previous section to evaluate the ability of rejecting overconfident predictions based on the mean predictive probability.

**Figure 6:**
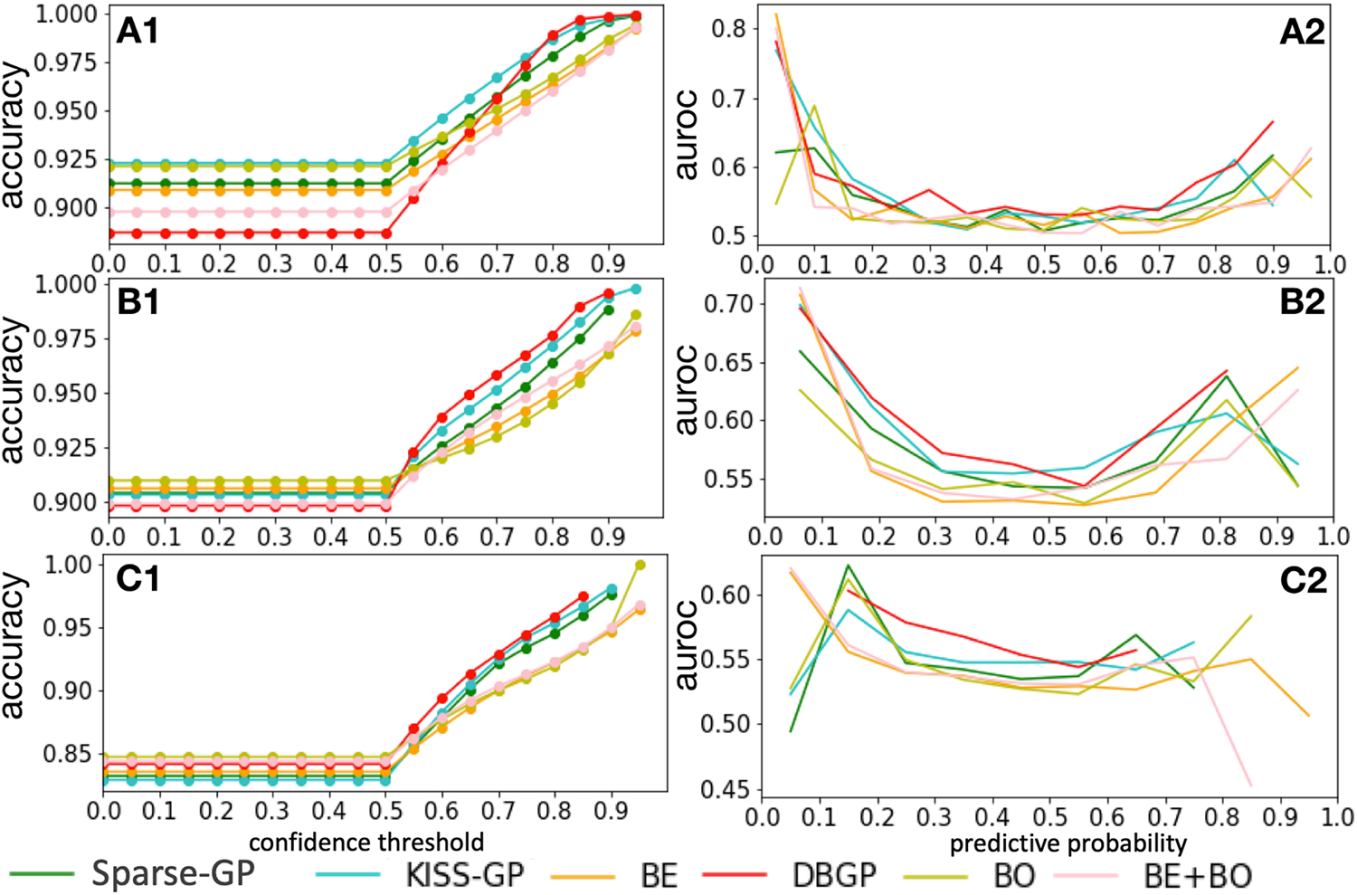
Accuracy and AUROC vs confidence curves. A: Heart failure, B: Diabetes, C: Depression. DBGP has higher accuracy in general and especially for predictions with high confidence, and it means DBGP is better at avoiding making overconfident predictions.

For the accuracy vs confidence curve, we treated the prediction as a two class classification task, with the mean prediction $p(y=k|x)$, where *k* represents the $k^{th}$ class, which is 2 in total in our case (binary classification). We defined the predicted label as ${\hat{y}} = argmax_{k}p(y=k|x)$ and the confidence as $p(y={\hat{y}}|x)=max_{k}p(y=k|x)$. The performance of accuracy for patients with confidence above different thresholds is shown in Fig. 6. A1, B1 and C1 are the evaluations for HF, diabetes, and depression, respectively. Because we only considered samples with confidence above each threshold, we would expect a rise in accuracy with increase in confidence thresholds. The figures show that the GP-based methods outperform the BDL models. Furthermore, the proposed method DBGP shows a better performance than the other models, especially for the high-confident predictions.

Considering that people are usually interested in predictions with a predictive probability higher than a given threshold, the accuracy curve measures the model performance in a more practical way. However, it also loses details of performance between positive and negative predictions. In this case, we further carried out an analysis to measure AUROC over different predictive probabilities as shown in A2, B2 and C2 in Fig. 6. These figures illustrate that the proposed DBGP model has a better AUROC over the predictive probability in general. Furthermore, it shows more robust predictions for highly confident predictions, especially for minority (positive) class. For example, in A1, B1, and C1, DBGP has relatively low accuracy comparing to other methods for low confidence thresholds. However, it outperforms other methods when the confidence threshold increases. This indicates that most of the misclassifications for DBGP are in the low confidence area, and its high confident predictions are more reliable than the other methods, which is a desired feature in practice. Additionally, instead of giving overconfident predictions, DBGP shows a better capability of penalizing highly confident predictions, and such effects become clearer when the model prediction performance drops (among HF, diabetes and depression prediction).

### Uncertainty estimation

In this experiment, we evaluated the performance of uncertainty estimation for models based on the aforementioned two criteria in Section : (1) predictions should intuitively have a higher model uncertainty for the minority class than the majority class in imbalanced datasets; and (2) the TP/TN predictions should have less predictive uncertainty than the FP/FN predictions.

**Figure 7:**
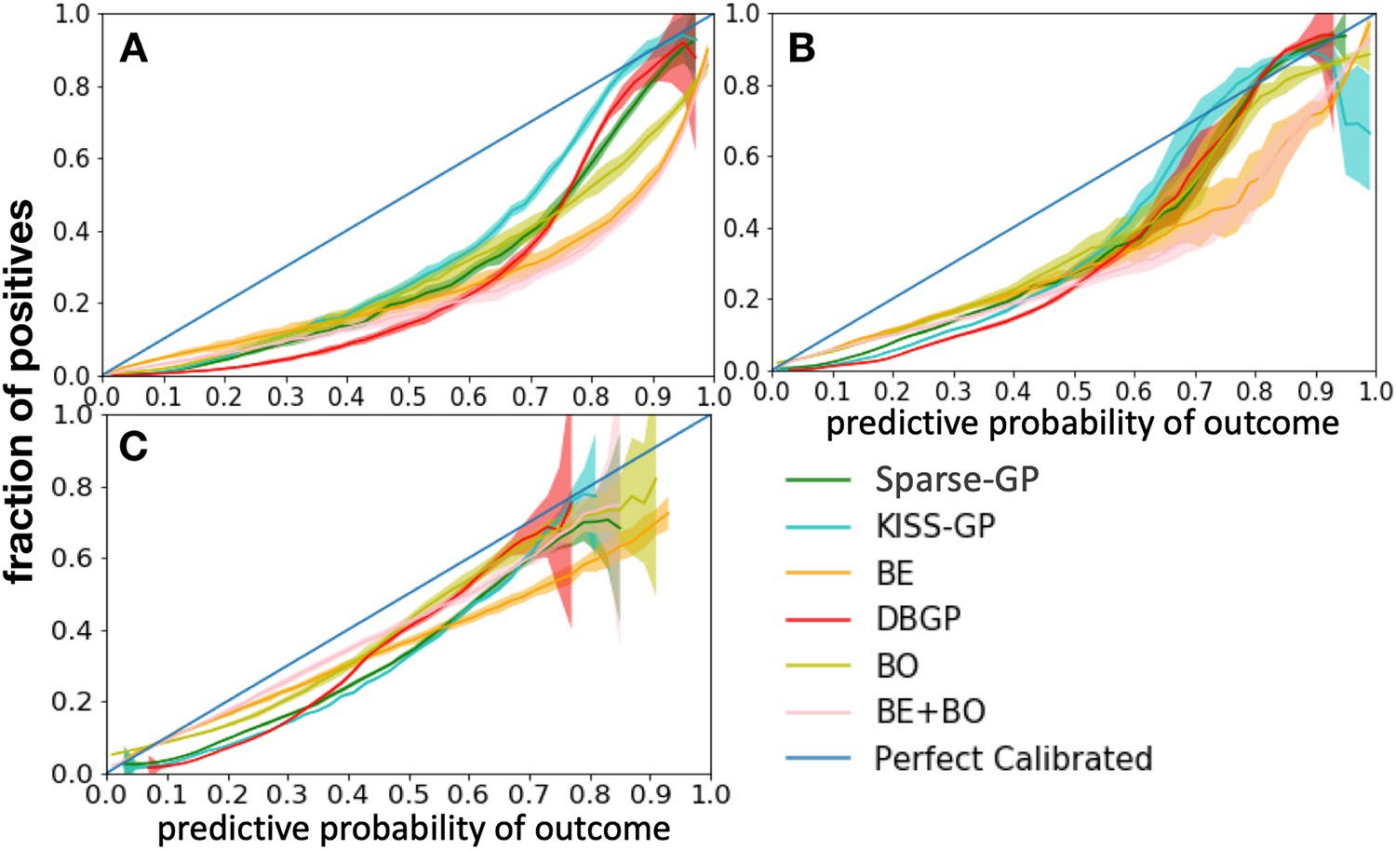
Calibration curve with 95% confidence interval. The x and the y axis represent the predictive probability and the fraction of positive cases, respectively; A: heart failure, B: diabetes, C: depression.

We firstly use a calibration curve to estimate the model uncertainty over predictive probability. By sampling weights from the posterior distribution, we consider each set of weights as an independent model and Fig. 7 shows the mean and 95% confidence intervals of calibration curve for the sampled models.

The uncertainty of a calibration curve reflects the uncertainty of a model (i.e., epistemic uncertainty) toward the prediction based on the data it is trained on. The uncertainty is caused by the uncertainty of the model parameters, moreover, it indicates the lack of knowledge (i.e., insufficiency of samples). It is different from the predictive uncertainty and it does not have the same relation as the predictive uncertainty and the confidence. As shown in Fig. 7, for BDL-based models, we can observe that for the HF prediction task, even though the prediction performance in terms of AP (around 0.6 as shown in Table 1) is relatively poor, they are still very certain (low confidence interval) for almost all positive predictions. The uncertainty only starts to show when the model performance is even worse, as shown in diabetes and depression prediction task in Table 1. In contrast, DBGP shows the capability of capturing high uncertainty for the positive predictions and indicates the insufficiency of positive cases for all three diseases. The potential reasons can be that the GP classifier makes inference based on the inducing points, and the inducing points are more sensitive to the data imbalance and are better at representing the incomplete coverage of the domain from the training samples than the stochastic linear classifier with a mean field variational distribution. Therefore, the GP-based methods are better at capturing uncertainties for minority class predictions in imbalanced datasets than the BDL-based models. Furthermore, because the diagonal line represents the perfect calibrated curve. We see that DBGP in general is closer to the line for the high-confidence positive predictions, thus, illustrating its better performance in terms of calibration for positive predictions. Additionally, the figure also shows the consistency with the previous results in Fig. 6, that the proposed method DBGP has a better rejection capability, and the rejection becomes clearer when the generalization performance drops. We see that when prediction performance drops, for example, depression has the worst performance in terms of AUROC and AP among these three tasks (Table 1). DBGP is able to avoid making high confidence predictions (in general with a predictive probability higher than 0.8 and lower than 0.1), especially for those high uncertain positive cases.

**Figure 8:**
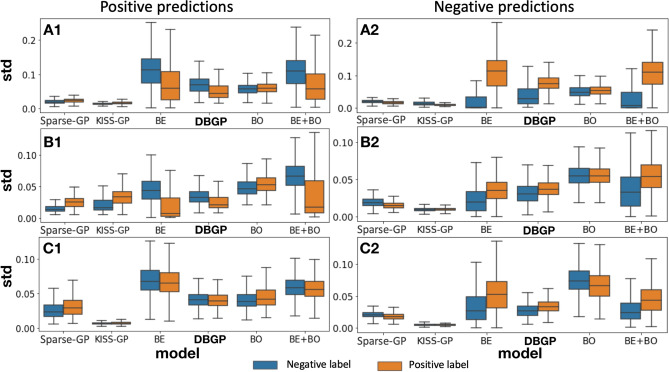
Distribution of std for positive and negative predictions. A, B and C represent the HF, diabetes and depression prediction tasks, respectively; 1 represents samples with positive prediction, and 2 represents samples with negative prediction, for instance: A1 means positive prediction for the HF prediction task. BDL-based methods as well as DBGP show the false positive and negative predictions have a higher uncertainty than true positive and negative predictions.

Additionally, to evaluate the uncertainty difference between TP/TN and FP/FN, we represented each patient with a mean predictive probability and a corresponding std calculated from samples from predictive distributions. Furthermore, we considered samples with predictive probability higher and less than 0.5 to be predicted as positives and negatives, respectively. Figure 8 uses the boxplot to show the distribution of std for all positive predictions and negative predictions. It shows that the Bayesian-embedding-based methods have better capability to capture the uncertainty to distinguish the TP/TN and FP/FN. On the contrary, the GP-based methods either provide an indistinguishable uncertainty estimation for TP/TN and FP/FN, or provide uncertainty estimations that seem incorrect, as shown by the uncertainty for TP/TN being even higher than the FP/FN. Therefore, for those models that show the correct trend, we propose to calculate the divergence between the distribution of TP/TN and the distribution of FP/FN to quantify the quality of distinguishability, as explained in Section . The results are shown in Table 2.

**Table 2:** Uncertainty divergence (DIV): DIV calculates the KL divergence between the std distributions of false positive and true positive predictions.

Model	HF		Diabetes		Depression	
	P	N	P	N	P	N
DBGP	0.330	0.867	0.105	0.146	0.009	0.279
BE	0.298	0.995	0.585	0.286	0.009	0.250
BE+BO	0.360	0.224	0.427	0.311	0.009	0.158

*P* positive prediction, *N* negative prediction.

Table 2 indicates that the uncertainty estimation is ambiguous for TP and FP if the model prediction performance is relatively poor, such as for depression. As the prediction performance improves (diabetes), the Bayesian methods become slightly better than DBGP. However, when the prediction performance reaches a certain level (HF), DBGP equally performs as well as the deep Bayesian models.

### Embedding analysis

The DBGP framework not only improves the ability of uncertainty estimation, but also brings a certain level of interpretability to the underlying deep architecture. In this section, we analyzed the uncertainty of diagnoses and medications by measuring the summation of entropy across all embedding dimensions. It can help to understand how the embedding affects the uncertainty in the latent representation. Entropy is a commonly used metric to quantify the uncertainty of a probability distribution^33^.

**Table 3:** Top 10 lowest entropy diagnoses/medications for heart failure, diabetes, and depression.

Entropy	HF
− 236.14	Diuretics
− 235.67	Acute myocardial infarction
− 235.43	Unspecified acute lower respiratory infection
− 235.17	Other ill-defined heart diseases
− 235.12	Antiplatelet Drugs
− 235.08	Chronic obstructive pulmonary disease with (acute) exacerbation
− 234.97	Chronic obstructive pulmonary disease, unspecified
− 234.95	Torticollis
− 234.90	Cellulitis and acute lymphangitis of other parts of limb
− 234.68	Type 2 diabetes mellitus with circulatory complications
Entropy	Diabetes
− 230.52	Drugs Used In Diabetes
− 230.28	Detection Strips, Urine For Glycosuria
− 229.18	Unspecified diabetes mellitus
− 229.16	Hypoglycemia, unspecified
− 229.00	Obesity, unspecified
− 228.42	Essential (Primary) Hypertension
− 227.24	Positive Inotropic Drugs
− 226.98	Lipid-Regulating Drugs
− 226.96	Chronic tubulo-interstitial nephritis, unspecified
− 226.77	Sex Hormones
Entropy	Depression
− 229.68	Antidepressant Drugs
− 221.35	Anxiety Disorder, Unspecified
− 220.69	Influenza due to unidentified influenza virus with other respiratory manifestations
− 219.65	Drugs Used In Psychoses and Rel.Disorders
− 219.58	Analgesics
− 219.36	Hypnotics And Anxiolytics
− 219.24	Laxatives
− 219.15	low back pain
− 219.13	General Anaesthesia/Hypnotics And Anxiolytics
− 219.09	Dyspep and Gastro-Oesophageal Reflux Disease

Because we only imposed the uncertainty to the embedding layer, all the uncertainty of the latent representation came from the embedding. Intuitively, a higher uncertainty of the embedding contributes more to the uncertainty of the latent representation. Therefore, if an embedding has a high uncertainty, it can mean a diagnosis or medication has more complex contextual information, and its association with the prediction is more unclear. In this case, such information can give us guidance to: (1) whether a causal or direct association is more likely to be included in the low uncertainty (low entropy) group; (2) whether the joint association has a higher chance to be included in the high uncertainty (high entropy) group. We list the diagnoses and medications with top 10 lowest entropy from HF, diabetes, and depression in Table 3.

Table 3 shows that 28 out of 30 of the low entropy diagnoses and medications are closely associated to the outcome. The results were validated by two clinicians and evidence from previous research can also support the linkage. For instance, Coughlin’s work^34^ and MChiro et al.’s work^35^ indicated a significant association between influenza virus, chronic low back pain, and anxiety and depression, respectively. However, there is no clear evidence for the association between torticollis, cellutitis, and heart failure. The method shows that most of the diagnoses and medications align with the prior knowledge and suggests a link between uncertainty and risk factor analyses. Therefore, it can be used to generate hypotheses for further clinical confirmation and causality analyses.

## Discussion

In general, we have proposed a mixed architecture named DBGP. It combines the strengths from both GPs and BDL, showing a comparable generalization performance with GPs and BDL-based probabilistic models in terms of AUROC, average precision and accuracy, but with a substantial better uncertainty estimation. In our experiments, with average precision only 0.62 to 0.65 for heart failure, 0.52 to 0.54 for diabetes, only GP-based methods show a clear pattern of having high model uncertainty for positive predictions (minority class) in highly imbalanced dataset; BDL-based methods are not sensitive to this information until the average precision become very low (0.40 to 0.43 for depression prediction). On the contrary, BDL-based methods are better at capturing the general trend for predictive uncertainty estimation. For instance, those methods can correctly show TP/TN predictions are more certain than FP/FN predictions, but patterns from GP-based methods do not match human’s intuition and expectation, having more certain predictions for FP/FN predictions than TP/TN predictions. By combining both methods, DBGP shows correct patterns in both ways, providing a better capability for uncertainty estimation. This can be used to indicate data insufficiency and guide further model improvement. It also can identify potential false negative predictions and resort those patients to seek medical check-ups to carry out more careful conclusions in practice rather than been ignored because of low risk prediction from risk model. Furthermore, we investigated the associations between uncertainty and risk factors, and the results showed a strong evidence for the relation. Therefore, an interesting topic for future work lies in an interpretability and causality analysis.

Our work also has restrictions, challenges, and limitations. First, to preserve patients with rich information for chronic condition prediction, we require patients to have more than 3 years of records and no less than 10 unique diagnosis codes in the medical history in cohort selection. This can potentially weaken its applicability in practice for patients with less or insufficient information. However, these restrictions can be relaxed to better fit the clinical question of interest in the future, and it does not compromise our proposed model architecture or uncertainty estimation. Additionally, the proposed model has overestimated risk for HF, diabetes, and depression risk prediction as shown in the calibration curves. From the clinical standpoint, a poorly calibrated risk prediction system can affect the clinical decision-making and limit its contribution to the intended area. However, calibration for rare disease risk prediction and in general for imbalanced dataset is a common challenge. Further investigations will be performed to explore potential techniques to mitigate miscalibration in our future work. Last but not least, our current work is only based on CPRD and it lacks ground truth to numerically evaluate how well the posterior is estimated as well as the rejection ability. Therefore, validate rejection ability or capability of handling distribution shift on other clinical datasets with different population and ways of data collection, and an evaluate of the posterior estimation of DBGP on simpler questions with ground truth are planed for future works.

## Data Availability

The data that support the findings are available from Clinical Practice Research Datalink (https://www.cprd.com). The accessibility of the data is clearly explained on the website: “Access to data from Clinical Practice Research Datalink is subject to a full licence agreement containing detailed terms and conditions of use. Patient level datasets can be extracted for researchers against specific study specifications, following protocol approval from the Independent Scientific Advisory Committee (ISAC) of UK.” Thus, the data for this study are under license, and are not publicly available.

## Code availability

Code associated with this study will be available upon acceptance of the manuscript.
