# Publisher Correction: Deep Bayesian Gaussian processes for uncertainty estimation in electronic health records

**DOI:** 10.1038/s41598-021-01680-x

**Published:** 2021-11-09

**Authors:** Yikuan Li, Shishir Rao, Abdelaali Hassaine, Rema Ramakrishnan, Dexter Canoy, Gholamreza Salimi-Khorshidi, Mohammad Mamouei, Thomas Lukasiewicz, Kazem Rahimi

**Affiliations:** 1 Deep Medicine, Oxford Martin School, University of Oxford, Oxford, United Kingdom; 2 Department of Computer Science, University of Oxford, Oxford, United Kingdom

Correction to: *Scientific Reports* https://doi.org/10.1038/s41598-021-00144-6, published online 19 October 2021

The Funding section in the original version of this Article was omitted. The Funding section now reads:

“The following authors are supported by grants from the British Heart Foundation (BHF): YL & KR (grant number: FS/PhD/21/29110) and KR & DC (grant number: PG/18/65/33872); KR is also in receipt of funding from the UKRI’s Global Challenges Research Fund (GCRF), Grant Ref ES/P0110551/1, Oxford NIHR Biomedical Research Centre and the Oxford Martin School (OMS), University of Oxford. The funders had no role in study design, data collection and analysis, decision to publish, or preparation of the manuscript. The views expressed are those of the authors and not necessarily those of the OMS, the BHF, the GCRF, the NIHR or the Department of Health and Social Care.”

The original Article has been corrected.

